# Antioxidant Metabolism, Photosystem II, and Fatty Acid Composition of Two Tall Fescue Genotypes With Different Heat Tolerance Under High Temperature Stress

**DOI:** 10.3389/fpls.2018.01242

**Published:** 2018-08-22

**Authors:** Lianlian Hu, Aoyue Bi, Zhengrong Hu, Erick Amombo, Huiying Li, Jinmin Fu

**Affiliations:** ^1^Key Laboratory of Plant Germplasm Enhancement and Specialty Agriculture, Wuhan Botanical Garden, The Chinese Academy of Sciences, Wuhan, China; ^2^University of Chinese Academy of Sciences, Beijing, China; ^3^The Institute for Advanced Study in Coastal Ecology, Ludong University, Yantai, China

**Keywords:** tall fescue, heat stress, antioxidant metabolism, photosystem II, fatty acid composition, gene expression

## Abstract

Tall fescue (*Festuca arundinacea Schreb*.) is a typical and widely used cool-season turf grass. High temperature is a key factor that limits its utility. The objectives of this study were to investigate the behaviors of fatty acid composition and its gene expression patterns in heat-resistant genotype “TF71” and heat-sensitive genotype “TF133” exposed to heat stress (40/35°C, 14/10 h), and to broaden our comprehension about the relationship between heat tolerance and fatty acid function. The result showed that heat stress increased the malondialdehyde (MDA) content and relative electrolyte leakage (EL), but decreased the level of chlorophyll and the activity of superoxide dismutase (SOD) and peroxidase (POD) when compared to the controls, to a greater extent in “TF133.” This result proved that “TF71” had superior high-temperature resistance. Furthermore, comparing the changes in the composition of fatty acid and the expression of the genes involved in its synthesis between the two different genotypes under heat stress, we found that heat stress increased the degree of unsaturation, UFA/SFA, and double bond index (DBI) in “TF71.” Moreover, quantitative RT-PCR revealed that heat stress altered the expression of the genes involved in fatty acid synthesis, including *ACAC*, *FabD*, *FabF*, *FabH*, *FabI*, and *FatA.* According to these findings, we can speculate that increasing the unsaturation degree of fatty acid or controlling the equilibrium ratio of UFA/SFA might be closely associated with the improving of the heat resistance in tall fescue.

## Introduction

Supra-optimal temperature is an important factor that limits the widespread use of cool-season turf grass in transitional and warm climatic regions, causes decline in turf quality, and leads to growth retardation and irreversible damage (Kotak et al., 2007). In addition, heat stress can induce a series of changes including enzyme activity, membrane fluidity, metabolism homeostasis, and genes transcription (Knight and Knight, 2001).

Plant cell membranes are one of the most sensitive parts during the perception of heat stress. The adaptability of cell membranes under high-temperature stress reflects the ability to adapt to adverse environmental conditions (Georgieva, 1999). Malondialdehyde (MDA) content and relative electrolyte leakage (EL) are effective indicators of the thermal stability of the cell membrane, and also considered as two effective indices to reflect the direct damage degree of heat stress on the plants. Many experiments have confirmed that high-temperature stress can cause the accumulation of reactive oxygen species (ROS) in plants, thereby causing membrane lipid oxidation, which is regarded as an oxidative stress (Liu and Huang, 2000; Huang et al., 2001). For self-protection against oxidative stress, plants up-regulate the activities of antioxidant enzymes such as superoxide dismutase (SOD) and peroxidase (POD) to scavenge ROS (Cushman and Bohnert, 2000).

Negative effects of heat stress on plants are largely due to their impact on photosynthesis. One of the most thermosensitive sites in the photosynthetic is considered to be photosystem II (PS II) and its activity decrease significantly under heat stress (Zhao et al., 2008). Many studies have shown that high temperature stress has three main effects on PS II function. First, high temperature induces a dissociation of the peripheral antenna complex of PSII from its core complex. In addition, high temperature leads to the deactivation and dissociation of the oxygen-evolving PS II complexes (Busheva et al., 2012). Furthermore, high temperature stress inhibits the electron transfer from the primary acceptor plastoquinone (Q_A_) to the secondary acceptor plastoquinone (Q_B_; Pospíšil and Tyystjärvi, 1999; Liu et al., 2002). The oxygen-evolving PS II complexes contain the intrinsic chlorophyll-binding proteins (CP43 and CP47; Pagliano et al., 2011). Overcoming photo damage to PS II is a rapid and efficient method for damage repair in photosynthetic organisms, which requires the *de novo* synthesis of the above-mentioned proteins (Yang et al., 2015).

Under heat stress, plants exhibit various mechanisms for surviving. They can tolerate high temperature by modifying the antioxidant system. For example, heat-resistant cool-season turf grass species had lower production of ROS compared to heat-sensitive species (Xu et al., 2006). Besides, photorespiration is a mechanism by which plants protect the photosynthetic apparatus against high temperature (Veste et al., 2000). Furthermore, in case of sudden heat stress, the changes in membrane lipid composition are very important for survival (Zabed and Shuichi, 2013), but its specific mechanism is not clear in turf grass under heat stress.

Previous studies have shown that the composition and saturation of lipids are closely associated with the plant ability to adapt to stress (Sui et al., 2010; Sanghera and Sharma, 2011). The total fatty acid content of plant was comprised of polyunsaturated fatty acids (PUFAs) mainly including linoleic acid (C18: 2) and linolenic acid (C18: 3), monounsaturated fatty acids (MUFAs) mainly including oleic acid (C18: 1), and the remaining is saturated fatty acids (SFAs) mainly including palmitic acid (C16: 0) and stearic acid (C18: 0; Mishra et al., 2015; Janila et al., 2016). Palmitic acid, stearic acid, linoleic, and linolenic acid are the four major fatty acids, as well as the major components of membrane lipids. Many experiments have confirmed that the unsaturation degree of fatty acids in cell membranes increased under supra-optimal temperature. The high level of fatty acids, especially unsaturated fatty acids (UFAs), can preferably maintain the fluidity of plant cell membrane lipids under stress (Peng et al., 2013), since the UFAs have double bonds, and more common double bonds to help to keep membrane fluidity (Vigh et al., 1998).

A series of enzymes including acetyl-CoA carboxylase, fatty acid synthase (FAS), fatty acid desaturase, and fatty acid elongase are involved in complex fatty acid biosynthesis pathways. The biosynthesis of long-chain fatty acids occurs in two different steps, i.e., the conversion of acetyl-CoA to malonyl-eoA, which is catalyzed by acetyl-CoA carboxylase. Subsequently, acetyl-eoA and malonyl-CoA are converted to palmitate in the presence of NADPH, which is catalyzed by the FAS (Wakil et al., 1983). FAS system is a prokaryotic multi-enzyme complex (type II FAS), which consists of an acyl carrier protein, an acyl acyltransferase, a malonyltransferase, a β-ketoacyl-ACP synthetase, a β-ketoacyl-ACP reductase, β-Hydroxyacyl-ACP dehydratase, and β-enoyl-ACP reductase (Lu, 2000). The process of linolenic acid biosynthesis is complicated and catalyzed by a battery of desaturase enzymes. First, stearoyl-ACP desaturase converts stearic acid to oleic acid (C18: l). Then C18: 1 is catalyzed by oleoyl-PC desaturase to form a linoleic acid (C18: 2). Finally, the formation of linolenic acid (C18: 3) is executed by linoleoyl-PC desaturase (Cyril et al., 2002).

Tall fescue (*Festuca arundinacea* Schreb.), a typical cool-season forage and turf grass species which grows in the temperate regions of the world. This turf grass has been widely cultivated for its major vegetative ground cover in landscape on account of its fast reproduction and strong resistance to drought, wear, and disease (Bush and Fannin, 2009). However, with global warming, the supra-optimal temperature becomes a key factor that limits tall fescues’ growth and utility. Our previous studies indicated that there was a great variation in heat tolerance among different tall fescue genotypes (Sun et al., 2014). However, up to date, there is limited information about the responsive differences among different genotypes under high temperature.

In this study, a heat-sensitive tall fescue genotype “TF133” and a heat-resistant genotype “TF71” were selected to investigate the different responses to high-temperature stress, and the possible mechanism involved in heat resistance in tall fescue. The membrane peroxidation, PS II photochemistry combined with fatty acid composition and gene expression patterns in response to heat stress were investigated between the two distinct tall fescue genotypes. This is the first study to demonstrate the heat resistance differences from the aspect of fatty acids. The study will provide useful insight into the relationship between the fatty acids function and heat tolerance.

## Materials and Methods

### Plant Materials

The study was conducted at Wuhan Botanical Garden, Chinese Academy of Sciences, Wuhan, China, in 2016. Two tall fescue genotypes heat-sensitive “TF133” and heat-tolerant “TF71” were used in this study. The seeds were sowed in plastic pots (13 cm in diameter and 15 cm in depth) filled with nutrient soil, and there were 20 pots of material (10 for each genotype). The pots were then kept in a greenhouse with a daily maximum/minimum temperature of 22/18°C at 14/10 h photoperiod for 40 days to establish the tall fescue plant. The plants were then watered daily and fertilized twice a week with a half-strength Hoagland nutrient solution (Hoagland and Arnon, 1937), and mowed at 6 cm above the sand surface.

Before the treatments, 20 pots of the plants were transferred into the incubators for pre-adaptation. The plants were grown under controlled conditions for 7 days [light/dark regime of 14/10 h at 22/18°C, relative humidity of 70%, photosynthetic photon flux density of (PAR) 360 μmol m^-2^ s^-1^] and were sub-irrigated every other day with full-strength Hoagland nutrient solution.

### Heat Treatments

After 7 days of pre-adaptation, each genotype of tall fescue was divided into two identical groups (five pots for one group) and transferred into two growth incubators with the same growth condition except for temperature, respectively. The temperature of one incubator was set at 22/18°C (day/night) (optimal temperature), while the other was set at 40/35°C (high temperature), respectively. High temperature (40/35°C)was selected according to the previous study (Zhao et al., 2015). Therefore, the plants were subjected into four treatment regimes and each had five pots: (i) “TF133” under optimum temperature (SCK); (ii) “TF71” under optimum temperature (TCK); (iii) “TF133” under high temperature (SH); and (iv) “TF71” under high temperature (TH). The heat treatment began immediately and the temperature rose to 40°C. After 1 day of treatment, the third fully expanded leaves of tall fescue were collected and stored at -80°C for physiological and metabolic assays. Meantime, the chlorophyll (chl) *a* fluorescence transient was recorded at 0 and 1 day after the treatment.

### Chlorophyll (chl) *a* Fluorescence Transient

Chl *a* fluorescence was determined by using a pulse-amplitude modulation (PAM) fluorometer (PAM 2500, Heinz Walz GmbH) with high time resolution (10 μs). Each measurement was repeated for at least five times. Subsequently, the leaves were accommodated in the dark for 30 min, and all measurements were taken using a saturating light intensity of 2000 μmol photons m^-2^ s^-1^. The strong light pulses inducted chl *a* fluorescence emission was measured subsequently and digitized between 10 μs and 300 ms (Korres et al., 2003). The OJIP transients were then analyzed by using the JIP-test as described by Chen et al. (2013).

### The JIP-Test

The JIP-test is a multi-parametric analysis of the OJIP transient according to Strasser et al. (2004). Chlorophyll fluorescence kinetics curve referred to the changing process from initial fluorescence intensity *F*_0_ to a maximal intensity *F*_P_, and the dark-adapted oxygenic photosynthetic organisms show a OJIP rise when illuminated with high intensity actinic light. A typical JIP-test included four phrases: O-J (0.05–5 ms), J-P (5–50 ms), and I-P (50–1000 ms). Chlorophyll fluorescence kinetics curve provides valuable information on the magnitude of stress effects on photosynthesis function, and those changes in photochemistry can be deduced by original fluorescence measurements in JIP-test which is based on the energy flux in biofilm (Ni et al., 2012).

### Crude Enzyme Extraction

To extract crude enzyme, about 0.2 g of fully expanded leaves were grounded by using ice-cooled mortar and pestle in 4 mL of 150 mM, pH 7.0 sodium phosphate buffer (pre-cooled at 4°C). The homogenate was transferred into 10 mL tubes and centrifuged for 20 min at 12,000 rpm at 4°C. The resulting supernatant was the crude enzyme for physiological assays.

### Determination of MDA Content

The MDA content was determined by the thiobarbituric acid (TBA) method according to previous reports (Hu et al., 2012; Fan et al., 2015). A 1 mL of crude enzyme solution was homogenized in 2 mL 0.5% (v/v) TBA and 20% (v/v) trichloroacetic acid. The mixture was incubated at 95°C for 30 min in a water bath, then cooled to room temperature (25°C), and centrifuged at 12,000 rpm for 10 min under 20°C. The absorbance of the supernatant was determined at 523 and 600 nm with a spectrophotometer (UV2600, UNIC, Shanghai, China). MDA content was calculated with the following formula:

$$\text{MDA}\;\left(\text{mol}{\text{.g}}^{-1}\;\text{FW}\right)\;\text{=}\;\left[\left(\text{OD532}-\text{OD600}\right)*\text{L}\right]\;/\left(1*\varepsilon *FW\right)$$

where L indicates the volume of the extract solution, l indicates the thickness of cuvettes, 𝜀 is the extinction coefficient of 155 mM^-1^cm, and FW is the fresh weight of the leaves.

### Quantification of Electrolyte Leakage (EL)

To determine the EL, about 0.15 g leaves were collected from the plants and washed three times with deionized water. Subsequently, the leaves were cut into 0.5 cm long fragments and placed in 50 mL centrifuge tubes filled with 25 mL deionized water. The test tubes were shaken for 24 h at room temperature and the initial conductivity (Ci) was determined by a conductance meter (JENCO-3173, Jenco Instruments, Inc., San Diego, CA, United States). Then the tube-fragments systems were autoclaved at 121°C for 15 min to completely release the electrolytes, and the conductivity of the incubation solution with killed tissues (Cmax) was measured after the solution had cooled down to room temperature (Bi et al., 2016). The relative EL was calculated by the following formula:

RelativeEL(%) = Ci/Cmax∗100%.

### Antioxidant Enzyme Activity

Activities of SOD and POD were determined according to methods described by Fan et al. (2015). SOD activity was measured by monitoring the inhibition of nitro blue tetrazolium (NBT) reduction with a spectrophotometer at 560 nm; 100 μL of crude enzyme solution was mixed into 2.9 mL reaction solution which consisted of 50 mM sodium phosphate buffer (pH 7.8), 60 μM riboflavin, 195 mM methionine, 3 μM ethylenediamine tetraacetic acid (EDTA), and 1.125 mM NBT; 3 mL reaction solution was regarded as the control. The mixture was illuminated under 4000 lx fluorescent lamp for 60 min for chromogenic reaction, and then transferred to darkness to stop the reaction. One unit of SOD activity was defined as the amount of enzyme that inhibits the NBT reduction by 50%. The activity of SOD was calculated with the following formula: SOD (U⋅g^-1^FW) = (A_CK_-A_treatment_)/(0.5 ^∗^A_CK_^∗^FW). FW is the fresh weight of the leaves. As for POD activity measurement, 50 μL crude enzyme solution was added into 2.95 mL reaction solution containing 100 mM sodium acetate-acetic acid buffer (pH 5.0), 0.25% guaiacol, and 0.75% H_2_O_2_. Absorbance at 460 nm was recorded at per minute interval for 3 min. A unit of POD activity was defined as the changes in absorbance at 460 nm per minute. The activity of POD was calculated with following formula: POD (U⋅g^-1^FW) = ΔA460/FW. FW is the fresh weight of the leaves.

### Fatty Acid Extraction and FAME Analysis

The plant leaves (0.3 g) were grounded into fine powder using a mortar and pestle with liquid N_2_. The tissue powder was transferred to test tubes. Total lipid was then extracted using the method described by Mishra et al. (2015). Three milliliters of extraction solution [chloroform: methanol: water (1: 2: 0.8; v/v/v)] were added to each sample. The samples were vortexed for 20 min at room temperature. Then 50 μL 2 mg/mL of heptadecanoic acid (C17: 0) was added as an internal standard.

The corresponding fatty acid methyl esters (FAMEs) of fatty acids were prepared by transmethylation (Kumari et al., 2012). Transmethylation was carried out by adding NaOH (1%, v/v in methanol; 1 mL) in a 15 mL tube which contained extracted lipid followed by heating at 55°C for 15 min. Thereafter methanolic HCl (5%, v/v; 2 mL) was added, and the mixture was further heated at 55°C for 15 min. Then 3 mL of the ultrapure water-hexane mixture (1:2, v/v) was added into the mixture solution. The derivative FAMEs were extracted with hexane three times, vacuumed dried, and finally dissolved in hexane (200 μL).

The FAMEs samples were analyzed on GC-MS (Agilent 7890A/5975C, Agilent Technologies, Palo Alto, CA, United States). In brief, 1 μL of the derivative solution was injected into a DB-5MS capillary (30 m × 0.25 mm × 0.25 mm, Agilent Technologies, Palo Alto, CA, United States). The total analysis time of GC-MS is nearly 80 min and the specific program is as follows: the initial temperature of GC oven was kept at 70°C for 2 min. Then increased to 230°C with 3°C min^-1^ increment and finally maintained at 230°C for 10 min. Thereafter, the temperature was increased to 270°C with 10°C min^-1^ increment and kept for 10 min. Helium was used as the carrier gas, and the flow rate was set at 1 mg⋅mL^-1^. The determination was carried out by electron impact ionization at 70 eV in the full scan mode (*m/z* 30–650). The samples were quantified against an internal standard (100 μg heptadecanoic acid), and the content of each fatty acid was expressed as a proportion of the total fatty acids present in the sample. The double bond index (DBI) was calculated by using the formula: index = (16: 1) + (18:1) + 2 [(16: 2) + (18: 2)] + 3 (18: 3) (Larkindale and Huang, 2004). Parentheses indicate the proportion of the total fatty acid amount, which was composed of each fatty acid species.

### Quantitative RT-PCR (Q-PCR) Analysis

Total RNA was isolated and purified by using Trizol reagent (Invitrogen, America). The first strand cDNA was synthesized from 2 μg of total RNA with oligo (dT) 12-18 primer using cDNA synthesis kit (Fermentas, Canada) according to the operation manual. Gene-specific primers for quantitative RT-PCR are listed in **Table 1**. When designing the primers, we blast the reference genome of tall fescue against with *Arabidopsis thaliana*, and select the peculiar part of the homologous gene of tall fescue as the starting point and end point of the amplified fragment. The *TUB* gene was used as the internal reference in the Q-PCR reaction. The program for Q-PCR was 94°C for 3 min, followed by 45 cycles of 94°C for the 20 s, 50–55°C for 20 s, then 72°C for 30 s, with a final elongation at 72°C for 7 min. The experiment was performed on a chromo4 real-time detection system (MJ Research, Cambridge, MA, United States) using SYBR Green I to produce a fluorogenic intercalating dye. The data were normalized with the relative efficiency of each primer pair.

**Table 1:** Primer sequences used for Q-PCR analyses.

Gene	Encoded		Primers sequences (5′–3′)
*TUB*		F	ATGCTTTCGTCTTATGCCC
		R	CTCTTGGTTTTGATGGTTGC
*psbA*	D1 protein	F	GTATTTATTATCGCCTTCATCG
		R	AGGACGCATAVVVAAACG
*psbB*	CP47	F	TAGGCGTAACGGTGGA
		R	AATATCTCGGAACAAGG
*psbC*	CP43	F	TAATACGGCTTATCCGAGTGAGTTT
		R	TCTTGCCAAGGTTGTATGTCTTTT
*ACAC*	Ctyl-CoA carboxylase	F	TCGTGTTGTTGTGAAGTCT
		R	TGTTCCATAAGCCGTAGTAG
*FabD*	Malonyltransferase	F	CAGGATGCTTCAGATGCT
		R	CATAGTTACCAGGACACAGA
*FabF*	β-Ketoacyl	F	ATGGTAAGCATCACAGTTCA
	-ACP synthetase II	R	AGCACTATCACAGAGGAATG
*FabH*	β-Ketoacyl	F	GATTGACAACCGAGTAGCA
	-ACP synthetase III	R	CCACAGTCCATTGAGAAGG
*FabI*	β-Enoyl	F	GAAGGAAGTGCTGGAGTAA
	-ACP reductase	R	TGAAGGAAGTTGCTGAGAC
*FatA*	Acyl-ACP thioesterase	F	ATGGAAGAGCACAATACACT
		R	GAGGAAGCAGAGGAGGAA


*F and R represent forward and reverse, respectively.*

### Statistical Analysis

Five biological replicates were used in all the experiments, all results were expressed as mean ± SE (standard error). The analysis of variance (ANOVA) was performed by using SPSS statistical software package (Ver.16.0, SPSS Inc., Chicago, IL, United States) and DPS v7.05. The graphs were produced using Origin 8.0 (Origin Lab Inc., Hampton, VA, United States). Means were separated using Duncan’s multiple range tests at *P* < 0.05 level of significance.

## Results

### Physiological Response to the Heat Stress

**FIGURE 1:**
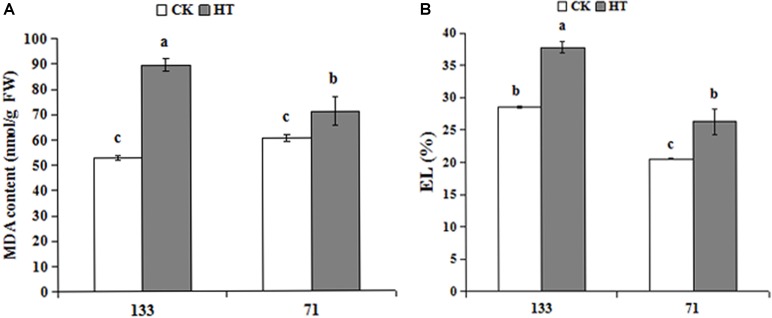
Changes of MDA content **(A)** and relative EL **(B)** in tall fescue leaves in heat-sensitive genotype “TF133” and heat-tolerant genotype “TF71” under normal conditions or heat stress. CK was the control (22/18°C for light/dark), while HT was the heat treatment (40/35°C for light/dark). All the plants were treated for 1 day. Mean ± SD were calculated from five biological repeats. Different letters indicated statistical difference significance at *P* < 0.05 among the different treatments groups by Duncan’s multiple range tests.

As shown in **Figure 1**, high temperature increased both MDA and relative EL in two tall fescue genotypes. Under heat stress, MDA contents were 69.1 and 17.3% higher for sensitive “TF133” and tolerant “TF71” when compared to their controls, respectively. Similar results were also observed regarding relative EL values. Relative EL in heat treated “TF133” and “TF71” was 32.5 and 27.9% higher than their respective controls.

### Changes in Antioxidant Activity Under Heat Stress

**FIGURE 2:**
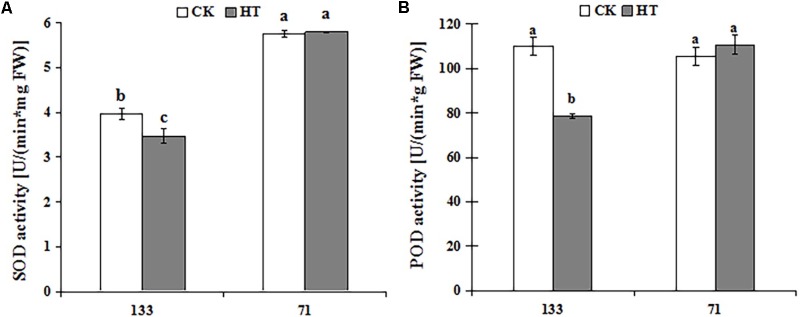
Changes of SOD **(A)** and POD **(B)** activities in tall fescue leaves in heat-sensitive genotype “TF133” and heat-tolerant genotype “TF71” under normal conditions or heat stress. CK was the control (22/18°C for light/dark), while HT was the heat treatment (40/35°C for light/dark). All the plants were treated for 1 day. Mean ± SD were calculated from five biological repeats. Different letters indicated statistical difference significance at *P* < 0.05 among the different treatments groups by Duncan’s multiple range tests.

There was a progressive decrease in SOD and POD activities during heat treatment for “TF133,” but no obvious change in “TF71” when compared to the control. As shown in **Figure 2**, high temperature dramatically decreased the activities of SOD and POD by 12.50 and 28.47% in “TF133,” respectively. It showed significantly different behaviors between heat-tolerant genotype and heat-sensitive genotype under the same high temperature.

### Changes in OJIP Transient Curves Under Heat Stress

**FIGURE 3:**
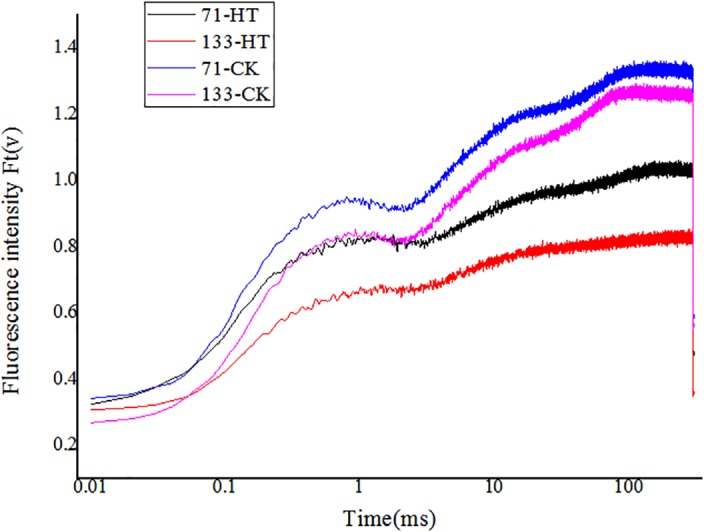
Alterations of chlorophyll fluorescence transients (OJIP curve) in tall fescue leaves in heat-sensitive genotype “TF133” and heat-tolerant genotype “TF71” under normal conditions or heat stress. CK was the control (22/18°C for light/dark), while HT was the heat treatment (40/35°C for light/dark). All the plants were treated for 1 day. Mean ± SD were calculated from five biological repeats. Different letters indicated statistical difference significance at *P* < 0.05 among the different treatments groups by Duncan’s multiple range tests.

High temperature significantly affected the OJIP fluorescence transient of both tall fescue genotypes. OJIP transient curves of control groups were higher than those of heat treatment groups (**Figure 3**). Furthermore, under normal condition, the OJIP transient curves exhibited higher levels for the heat-tolerant genotype versus the heat-sensitive genotype. High temperature led to more difference in OJIP between two genotypes.

To further investigate the structural alteration, functional parameters, and photosynthetic behaviors in different tall fescue genotypes under heat stress, the JIP-test was applied to analyze the value of OJIP transient curves. Basic fluorescence parameters including *F*_O_, *F*_K_, *F*_J_, *F*_I_, *F*_M_, and *M*_0_ were extracted (**Table 2**). Both genotypes generally had a higher level of above basic parameters for under control regimes versus high temperature regimes, except *F*_O_ and *M*_0_ which were lower under normal condition.

**Table 2:** Photosynthetic parameters deduced by the JIP-test analysis of fluorescence transients.

	C		H		Definitions
	′TF71′	′TF133′	′TF71′	′TF133′	
**Data extracted from the recorded OJIP fluorescence transient curves**				
*F*_0_ = *F*_20__μs_	0.35a	0.28c	0.35a	0.31b	Fluorescence at time t after onset of actinic illumination
*F*_K_	0.84a	0.74b	0.75b	0.59c	Fluorescence value at 300 μs
*F*_J_	0.91a	0.82b	0.79c	0.66d	Fluorescence value at the J-step (2 ms) of OJIP
*F*_I_	1.23a	1.15b	0.96c	0.78d	Fluorescence value at the I-step (30 ms) of OJIP
*F*_P_ = *F*_M_	1.39a	1.31b	1.08c	0.87d	Fluorescence value at the peak of OJIP test
*M*_0_	1.88b	1.81b	2.34a	2.00b	Approximate value of the initial slope of fluorescence transient curves
**Specific energy fluxes(per active PS II reaction center)**				
ABS/RC	4.65b	4.31c	5.39a	4.78b	Absorbed photon flux per RC
TPo/RC	3.56a	3.40b	3.60a	3.05c	Trapped excitation flux (leading to Q_A_ reduction) per RC
ETo/RC	1.59a	1.62a	1.30b	1.19b	Electron transport flux (further than Q_A-_) per RC
REo/RC	0.57a	0.56a	0.55a	0.48a	Electron transport reducing end electron acceptors at the PSI acceptor side, per RC
**Quantum yields and efficiencies/probabilities**				
φpo = TRo/ABS	0.74b	0.79a	0.68c	0.64d	Maximum quantum yield for primary photochemistry, namely *F*_V_/*F*_M_
öEo = ETo/ABS	0.34a	0.37a	0.25b	0.24b	Quantum yield of the electron transport flux from Q_A_ to Q_B_
δRo = RE0/RC	0.36ab	0.33b	0.41ab	0.46a	Quantum yield for reduction of end electron acceptor at the PSI acceptor side
γRC	0.18ab	0.19a	0.16b	0.17b	probability that a PS II Chl molecule functions as RC
**Performance Indexes (PI, combination of parameters)**				
PI_ABS_	0.51b	0.84a	0.20c	0.22c	PI(potential) for energy conservation from exciton to the reduction of intersystem electron
PI_]total_	0.31b	0.43a	0.21c	0.14d	PI(potential) for energy conservation from exciton to the reduction of PS I end acceptors


*The calculation of each parameter follows the method of Yusuf et al. (2010). Subscript “0” indicates that the parameter refers to the onset of illumination. Values are given as the average of five replicates, and different letters indicated statistical difference significance at *P* < 0.05 among the different treatments groups by Duncan’s multiple range tests.*

Marked differences in ABS/RC, TPo/RC, and ETo/RC were observed between control and high-temperature regimes. High temperature increased ABS/RC, but decreased ETo/RC for both tall fescue genotypes. In addition, TPo/RC was decreased just in heat-treated “TF133” plants, compared to non-treated plants. There were no differences in REo/RC between control and stress regimes for both genotypes. Parameters associated with quantum yield and efficiencies included φpo, φEo, Ro, and [scale=0.5]img001RC. Heat stress dramatically declined the value of φpo and φEo in both genotypes. In addition, it was noticed that the φpo value in tolerant genotype was significantly higher than in sensitive genotype when grown under normal conditions. Similar results were also observed for PI_ABS_ and PI_total_. There was no distinct difference in δRo and [scale=0.5]img001RC between two genotypes either under normal condition or heat stress. However, high temperature increased the δRo whereas reduced the [scale=0.5]img001RC values in both tall fescue genotypes.

### Changes in Fatty Acid Composition Under Heat Stress

**FIGURE 4:**
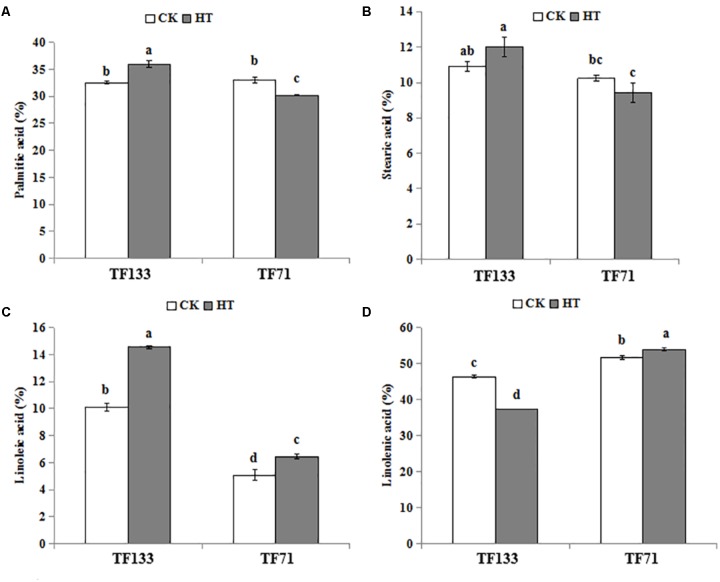
Changes of four major fatty acids in tall fescue leaves in heat-sensitive genotype “TF133” and heat-tolerant genotype “TF71” under normal conditions or heat stress. **(A)** Palmitic acid. **(B)** Stearic acid. **(C)** Linoleic acid. **(D)** Linolenic acid. CK was the control (22/18°C for light/dark), while HT was the heat treatment (40/35°C for light/dark). All the plants were treated for 1 day. Mean ± SD were calculated from five biological repeats. Different letters indicated statistical difference significance at *P* < 0.05 among the different treatments groups by Duncan’s multiple range tests.

Four major fatty acids were identified and quantified by using GC-MS in this study. There were two SFAs and two UFAs, i.e., palmitic acid (C16: 0), stearic acid (C18: 0) and linoleic acid (C18: 2), linolenic acid (C18: 3), respectively. As shown in **Figure 4**, the palmitic acid content was 32.21 and 33.0% in “TF133” and “TF71” under control condition, respectively. However, after heat treatment, the percentage was increased to 35.99% in “TF133” but decreased to 30.22% in “TF71” (**Figure 4A**). High temperature modestly increased the stearic acid content from 10.92 to 12.00% in “TF133” and decreased the stearic acid accounted 10.24 to 9.42% of total fatty acid content in “TF71” after heat treatment (**Figure 4B**). Under high temperature, the linoleic acid content was significantly increased from 5.08 to 6.45 and 10.12 to 14.55% in “TF71” and “TF133,” respectively (**Figure 4C**). Under control condition, the linolenic acid content was relatively higher in “TF71” than in “TF133.” After heat stress, however, the linolenic acid level was significantly declined from 46.33 to 37.38% in “TF133,” while moderately increased from 51.68 to 53.92% in “TF71” (**Figure 4D**).

**FIGURE 5:**
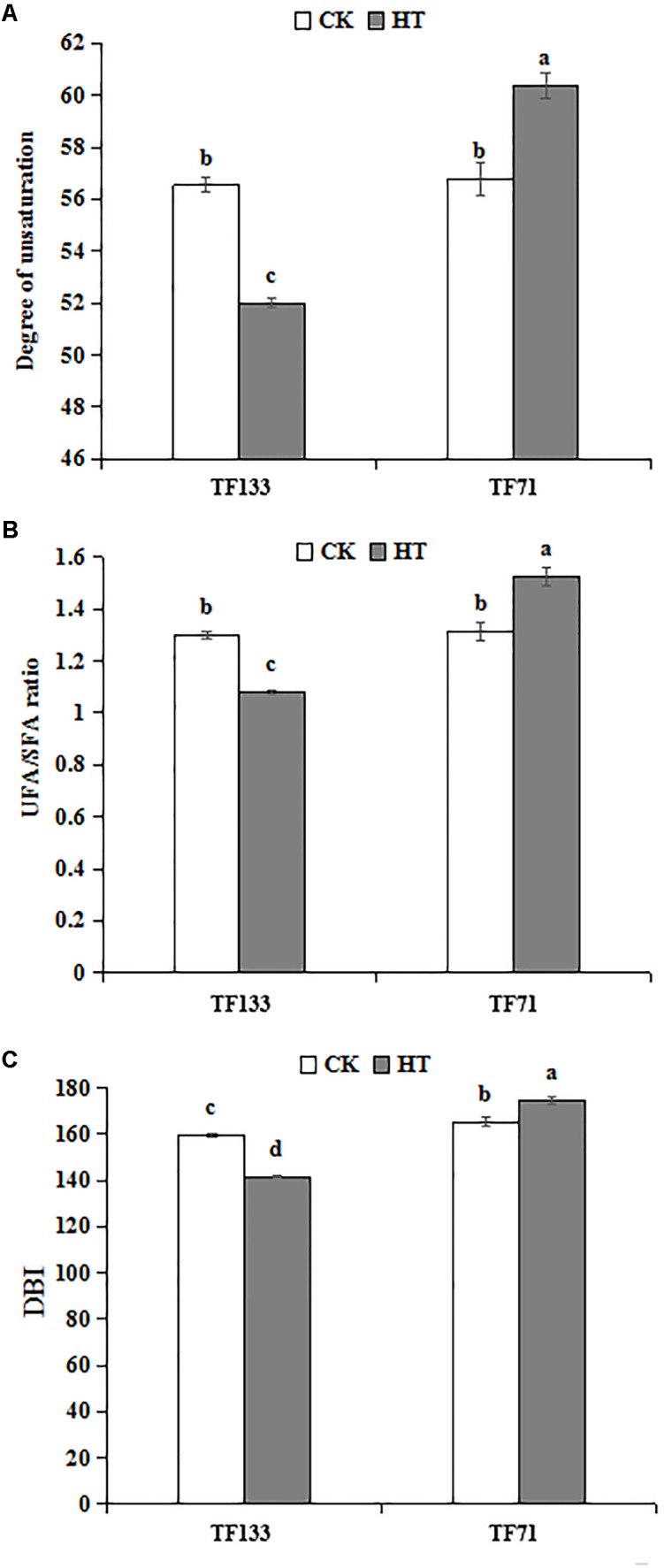
Changes of degree of unsaturation **(A)**, the ratio of UFA to SFA **(B)**, and double bond index (DBI) **(C)** in tall fescue leaves in heat-sensitive genotype “TF133” and heat-tolerant genotype “TF71” under normal conditions or heat stress. CK was the control (22/18°C for light/dark), while HT was the heat treatment (40/35°C for light/dark). All the plants were treated for 1 day. Mean ± SD were calculated from five biological repeats. Different letters indicated statistical difference significance at *P* < 0.05 among the different treatments groups by Duncan’s multiple range tests.

Under control condition, there was no difference in unsaturation degree of fatty acids between the two genotypes. However, after heat stress, the unsaturation degree was significantly decreased to 52.01% in “TF133” but increased to 60.37% in “TF71” (**Figure 5A**). To determine the fatty acid composition, the UFA/SFA ratio was determined. Under control condition, no obvious difference in UFA/SFA ratio was observed between the two genotypes. However, after heat treatment, the ratio decreased by 16.8% in “TF133,” while increased by 16.0% in “TF71” (**Figure 5B**), when compared to their controls, respectively. As for DBI, high temperature significantly decreased it by 11.43% for “TF133” but increased by 5.7% in “TF71” (**Figure 5C**).

### Expression Profiles of Genes Related to Photosynthetic System and Fatty Acid Synthesis Pathway

**FIGURE 6:**
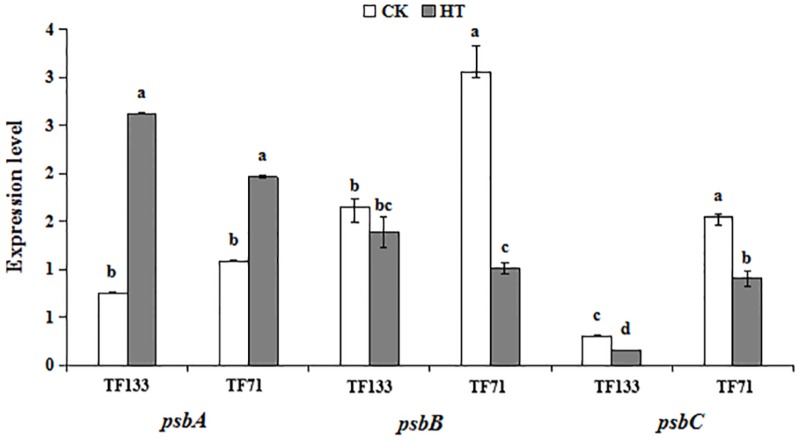
The effects of heat treatments on gene expression of *PsbA*, *PsbB*, and *Psbc* in tall fescue leaves in heat-sensitive genotype “TF133” and heat-tolerant genotype “TF71” under normal conditions or heat stress.CK was the control (22/18°C for light/dark), while HT was the heat treatment (40/35°C for light/dark). All the plants were treated for 1 day. Mean ± SD were calculated from five biological repeats. Different letters indicated statistical difference significance at *P* < 0.05 among the different treatments groups by Duncan’s multiple range tests.

**FIGURE 7:**
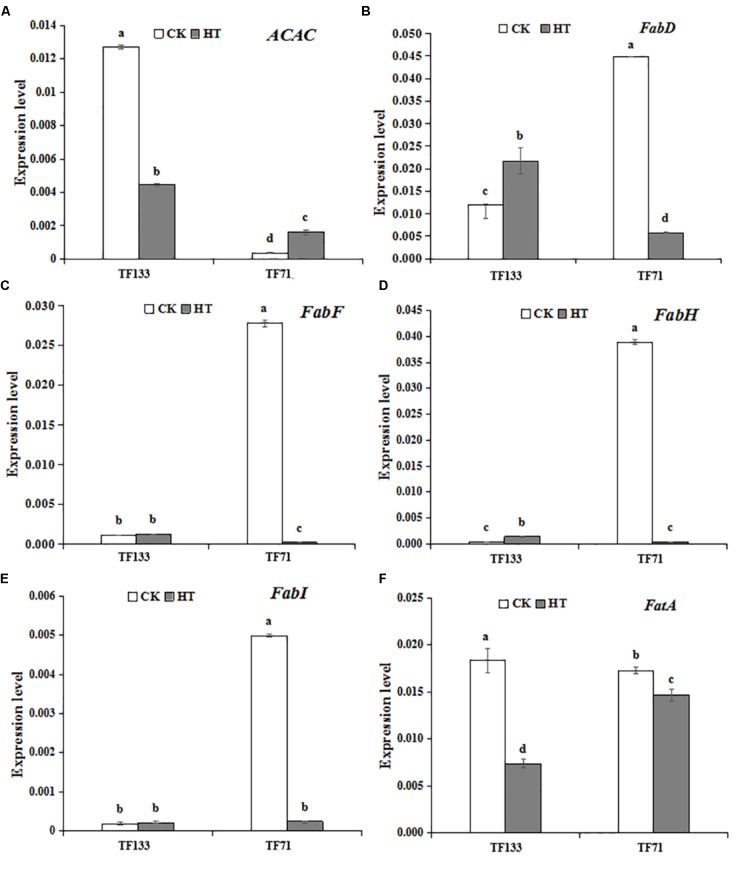
The effects of heat treatments on gene expression of **(A)** *ACAC* (encoding fore actyl-CoA carboxylase), **(B)** *FabD* (encoding fore malonyltransferase), **(C)** *FabF* (encoding fore β-ketoacyl-ACP synthetase II), **(D)** *FabH* (encoding fore β-ketoacyl-ACP synthetase III), **(E)** *FabI* (encoding fore β-enoyl-ACP reductase), and **(F)** *FatA* (encoding fore acyl-ACP thioesterase) in tall fescue leaves in heat-sensitive genotype “TF133” and heat-tolerant genotype “TF71” under normal conditions or heat stress. CK was the control (22/18°C for light/dark), while HT was the heat treatment (40/35°C for light/dark). All the plants were treated for 1 day. Mean ± SD were calculated from five biological repeats. Different letters indicated statistical difference significance at *P* < 0.05 among the different treatments groups by Duncan’s multiple range tests.

To investigate the gene expression pattern of photosynthetic system genes and fatty acid synthesis pathway in response to heat stress, three genes involved in the photosynthetic system and six genes involved in fatty acid synthesis pathway were analyzed by Q-PCR. High temperature significantly enhanced the gene transcription level of *PsbA* compared to the control regime in both tall fescue genotypes (**Figure 6**). After heat treatment, the transcription level of *PsbB* showed no obvious difference in “TF133,” but a remarkable decrease in “TF71,” when compared to their controls, respectively. The transcription level of *PsbC* was reduced by heat stress in both genotypes. In addition, the abundance of *PsbC* was higher in “TF71” than “TF133” under both control and heat stress conditions. The expression of *FabD* was up-regulated by heat treatment in “TF133,” but down-regulated in “TF71” (**Figure 7**). The similar trend was also observed for gene *FabH*. In contrast, *ACAC* displayed down-regulation in “TF133,” but up-regulation in “TF71” after heat treatment, when compared to their respective controls. The expression of both *FabI* and *FabF* was significantly inhibited by high temperature in “TF71,” whereas it showed no difference in “TF133,” when compared to their controls, respectively. In addition, the transcription level of *FatA* significantly declined in both genotypes under heat treatment.

## Discussion

High temperature is one of the most detrimental environmental stresses, which can induce cell damage and constrain plant growth. The excess generation of ROS is one of the major consequence of heat stress, which subsequently induces cell membrane injury, damage the photosynthesis systems and PSII oxygen evolving complex and influence the protein synthesis (Sairam et al., 2000; Wahid and Close, 2007). Plant can tolerate heat stress by physical changes within the plant body and by creating signals for changing metabolism (Mirza et al., 2013). Cell membrane injury is related to the composition and content of fatty acids in the lipid bilayers of the membrane (Yordanov et al., 2000; Gigon et al., 2004). However, the relationship between the composition and saturation level of fatty acids and heat tolerance in different plant genotypes is still unknown.

The photosynthetic activity of chloroplast is considered to be the most thermo-sensitive cell function. In this study, high temperature declined the OJIP transient curve level at both tall fescue genotypes, and to a greater extent in “TF133.” Abundant information can be revealed by the OJIP fluorescence transient, and it was used to determine the parameters through JIP-test (Krause et al., 2014). The *F_0_* is the level of fluorescence emission when all the primary quinone acceptors (Q_A_) were in oxidized state after dark adaptation. The *F_0_* rise of leaves results from the physical separation of the PS II reaction centers from their associated pigment antennae under heat stress, resulting in blocked energy transfer to the PS II traps and a decrease of the quantum efficiency of PS II (Briantais et al., 1996; Krause et al., 2014). The total performance index PI_total_ is the most general parameter in the JIP-test under stress conditions. It reflects the changes in intersystem electron and the energy conservation from exciton to the reduction of PSI end acceptors (Yusuf et al., 2010; Stefanov et al., 2011). The parameters φpo, öEo, and δRo were used to assess the impairment of components in PSII and investigate more details (Chen et al., 2013). The φpo and öEo values were remarkably changed while the δRo was slightly altered under heat stress, and the treatment with high temperature significantly decreased the behavior of φpo and öEo in tall fescue. Thus, the results suggested that the behaviors of PS II on both electron donor side and acceptor side are blocked under heat stress and PSI was less damaged than PSII (Apostolova and Dobrikova, 2010; Zushi et al., 2012). Furthermore, PI_total_ and PI_ABS_ were higher in non-treated than heat-treated plants, and to a higher extent in “TF133.” This result confirmed that in order to protect the plant from the harsh environmental condition, excess excitation energy was transformed into heat dissipation so as to keep the balance between energy absorption and utilization (Perales-Vela et al., 2007).

The alteration of photosynthesis was also related to the changes in gene expression patterns. CP47 (*psbB* encoded protein) and CP43 (*psbC* encoded protein) are the intrinsic transmembrane proteins, which located in the reaction center of PS II. Previous studies showed that the light harvesting antenna would be decoupled from the RC, and the damaged D1 protein would be cleavaged under high temperature (Yoshioka et al., 2006). Both of the transcription and translation levels of *psbA* will decrease under heat stress, meanwhile reducing the transcription product mRNA of *psbA* and D1 protein content (Yang et al., 2013). Moreover, the decrease of CP43 and CP47 would lead to a reduction in active RC and result in inefficient energy utilization (Vani et al., 2001). In this study, the expression of *psbA*, which encoding for D1 protein in the core of RC, is higher in heat-treated plants than in the controls. Its up-regulation is favorable for PSII RC against high temperature and confirmed that heat stress improves the susceptibleness of this photosynthetic organs (Takahashi et al., 2004).

Malondialdehyde and EL were valid indicators to show the degree of cellular injury caused by environmental stress (Hernández and Almansa, 2002). Furthermore, MDA is a final decomposition product of lipid peroxidation (Monteiro et al., 2008). The current results suggested that high temperature induced lipid peroxidation and plasma membrane permeability increases. The dismutation of converting O_2_^-^ to H_2_O_2_ is important in defense ROS, which is catalyzed by SOD. Then H_2_O_2_ was removed by POD to regulate the relatively stable level of H_2_O_2_ (Mansoor, 2013). In the present study, the activities of both SOD and POD in “TF71” are higher than “TF133,” suggested that the induction of antioxidant enzyme activities is closely contact with increased environmental stress (Ashraf, 2009) and increasing antioxidant enzyme activity to scavenge ROS will enhance plant heat resistant. In summary, the abilities of “TF71” is better than “TF133” in maintaining normal photosynthesis and cell membrane stability to protecting heat stress.

To further explore the possible heat-resistance mechanism underlying fatty acid, the lipid composition was analyzed in this study. Under heat stress, “TF71” maintained a relatively high level of UFA and was less affected by the thermal damage. It may be a protective mechanism in “TF71” to adapt to heat stress. The results of fatty acid composition indicated that unsaturated membrane lipid played an important role in improving plant heat resistance (Alvarezordóñez et al., 2008; Wang et al., 2013). DBI is an indicator for evaluating unsaturation level of fatty acids (Zhong et al., 2011). Under heat condition, the value of DBI declined in “TF133,” while it increased in “TF71.” This trend of “TF133” under heat stress is consistent with the change in *Arabidopsis* at 30°C (Li et al., 2015). Similar results were observed in the parameters of UFA/SFA ratio and degree of unsaturation. These results further suggested that relatively higher unsaturation degree of total lipid may contribute to the greater heat resistance in “TF71.”

The expression pattern of fatty acid synthesis related genes was also investigated. It is the carboxylation of acetyl-CoA that limited the rate of fatty acid synthesis. So that the transcription of acetyl-CoA carboxylase (*ACAC* encoded a protein) is closely linked with the later fatty acid synthesis. Malonyltransferase (*FabD* encoded a protein) is a key enzyme for the synthesis of fatty acids. It is a thiolase that catalyzes the initial step of the fatty acid synthesis, catalyzing the formation of malonyl-ACP with malonyl-CoA and ACP as substrates (Prigge et al., 2003). Malonyl-ACP plays an important role in the synthesis of type II fatty acids, which is an important substrate for the synthesis of palmitic acid (Eckhardt et al., 2013). β-ketoacyl-ACP synthetase II (KAS II) (*FabF* encoded a protein), KAS III (*FabH* encoded a protein), and β-enoyl-ACP reductase (*FabI* encoded a protein) are involved in fatty acid elongation. At present, there are three kinds of β-ketoacyl-ACP synthetase (KAS), which is KAS I, KAS II, and KAS III. KAS I catalyzes the condensation of 4 carbon to 14 carbonated acyl-ACP; KAS II catalyzes the condensation of 14 carbon and 16 carbonated acyl-ACP and determines the ratio of (16 carbon fatty acid) / (18 carbon fatty acid), while KAS III catalyzes the condensation of malonyl-ACP with acetyl-ACP and the subsequent one to two cycles. β-enoyl-ACP reductase catalyzes the last step of each cycle and generates an acyl-ACP with a 2-base increase (Lu, 2000).

In the present study, under normal condition, the transcription of *ACAC* in “TF133” is much higher than that in “TF71.” Meanwhile, the genes involved in long-chain fatty acid synthesis show an opposite trend. After heat stress, the transcription of *ACAC* decreased in “TF133” but still maintained a relatively high transcription level compared to that in “TF71,” even though the transcription in TF71 increased significantly. Then, the transcription of fatty acid synthesis genes has no significant change in “TF133,” but the transcription in “TF71” decreased to the same level with “TF133.” The results indicated that plant regulate the transcriptional balance between substrates synthesis and fatty acid synthesis to maintain a stable fatty acid content under different environment. Specific regulation is just like that keeping a lower transcription level of fatty acid synthesis genes when substrate is abundant, and maintaining a higher transcription when the substrate is low. This transcriptional regulation result in no significant change in SFA under heat stress in both genotypes. The results also showed to some extent that the relationship between fatty acid content and heat resistance is not as close as fatty acid composition.

Acyl-ACP thioesterase A (*FatA* encoded a protein) catalyzes the termination of FAS cycling. It determines the length and saturation level of free fatty acid transport in and out of plant plastids. In plant cell, SFA are catalyzed by the type II FAS system in the plasmid, and the final products are only C16: 0-ACP and C18: 0-ACP. Most C18: 0-ACP produced by elongation is desaturated by the stearoyl-ACP desaturase. The resulting C18: 1-ACP can enter the prokaryotic glyceroipid pathway or be hydrolyzed by *FatA* for export from the plastid (Vigh et al., 1993; Salas and Ohlrogge, 2002; Libeisson et al., 2010). The C18:1 diffused from plastid was transported to the endoplasmic reticulum and then be synthesized other glycerides. In the present study, the transcription of *FatA* significantly reduced in “TF133” after heat stress. This may cause a reduction in the hydrolysis and release of C18: 1-ACP, further affect the membrane lipid glyceride synthesis. The changes of PUFA under high temperature need to be further studied. According to the research results in *Arabidopsis* under high temperature of Li, the possible reason for this change is that the ER-based ω-6 desaturase responsible for the conversion of C18:1 to C18:2 was up-regulated at high temperature, concurrent with decreased proportion of C18:3 (Li et al., 2015).

In brief, the degree of membrane unsaturation is one determining factor in adaptation to high temperature stress. Our results in this study highlight the significance of changes in fatty acid composition under heat stress. At high temperature, tall fescue controls the equilibrium rate of substrate synthesis and fatty acid synthesis, so as to maintain the stability of fatty acid content while up-regulating the UFA to maintain good fluidity in “TF71.” Furthermore, this regulation helps to maintain the normal physiological characteristics of organisms in “TF71” and may contribute to the greater heat resistance.

## Conclusion

The mechanism in tall fescue response to heat stress is complex. Superior high-temperature resistance in “TF71” might be attributed to its better response to antioxidant metabolism, PS II, and improved proportion of UFA or its controlling the equilibrium ratio of UFA/SFA. Together, they were jointly conducive to membrane stability, and therefore enhanced the adaptation to high temperature in tall fescue.

## Author Contributions

JF and HL designed the research. LH carried out the experiments, analyzed the data, and wrote the manuscript. AB and ZH assisted with doing the experiments. JF, HL, and EA helped to draft the manuscript and revise the manuscript. All authors read and approved the final manuscript.

## Conflict of Interest Statement

The authors declare that the research was conducted in the absence of any commercial or financial relationships that could be construed as a potential conflict of interest.
